# Complement inhibitor therapy in thymoma-associated myasthenia gravis: a real-world experience

**DOI:** 10.3389/fimmu.2025.1562419

**Published:** 2025-04-14

**Authors:** Sofia Marini, Carmen Erra, Laura Fionda, Silvia Falso, Elena Rossini, Federico Habetswallner, Elisa Meacci, Martina Marini, Francesco Habetswallner, Raffaele Iorio

**Affiliations:** ^1^ Department of Neuroscience, Università Cattolica del Sacro Cuore, Rome, Italy; ^2^ UOC Neurophysiopathology, AORN Cardarelli, Naples, Italy; ^3^ Neuromuscular and Rare Disease Centre, Sant’Andrea Hospital, Rome, Italy; ^4^ Department of Neuroscience, Mental Health and Sensory Organs (NESMOS), Faculty of Medicine and Psychology, Sapienza University of Rome, Rome, Italy; ^5^ Department of General Thoracic Surgery, Fondazione Policlinico Universitario Agostino Gemelli IRCCS, Rome, Italy; ^6^ Neurology Unit, Fondazione Policlinico Universitario Agostino Gemelli IRCCS, Rome, Italy

**Keywords:** thymoma, myasthenia gravis, complement inhibitors therapy, real-world, neuromuscular disease, neuroimmunology

## Abstract

**Introduction:**

Thymoma-associated myasthenia gravis (TAMG) accounts for 15–20% of all myasthenia gravis (MG) cases and is typically characterized by severe clinical manifestations and suboptimal response to conventional therapies. However, TAMG patients are underrepresented in clinical trials, leaving gaps in evidence for optimal treatment strategies. This study assessed the efficacy of complement inhibitors (CI) in TAMG population.

**Methods:**

We retrospectively reviewed 23 TAMG patients who received CI, with a minimum follow-up of six months. Additionally, we randomly included 22 MG patients without thymoma, treated with CI, in the control group. Clinical outcomes were measured using Myasthenia Gravis-Activities of Daily Living (MG-ADL) and Quantitative Myasthenia Gravis (QMG) scores at baseline, three, and six months.

**Results:**

Among the 23 TAMG patients, 21 initiated CI after thymectomy, with a median interval of eight years (IQR:2.5-15) post-surgery. Two patients achieved sufficient stabilization on CI to undergo thymectomy thereafter. The most frequent thymoma histological subtype was WHO type B2, detected in 43.5% of cases. Median MG-ADL score decreased from 11 (IQR:8-15) to 3 (IQR:2-5) and 4 (IQR:1-5) at three and six months, respectively (both p<0.001). Median QMG score decreased from 16 (IQR:14-22) to 10 (IQR: 5-11) at three and six months (both p<0.001). Prednisone dosage was tapered in 20 patients. No significant differences were observed between TAMG and MG patients without thymoma in MG-ADL, QMG and steroid reduction.

**Conclusion:**

CI demonstrated significant improvements in MG-ADL and QMG scores, along with a steroid-sparing effect, suggesting its potential as an effective treatment for this challenging subpopulation.

## 1 Introduction

Thymoma-associated myasthenia gravis (TAMG) accounts for 15–20% of all myasthenia gravis (MG) cases and is typically associated with autoantibodies targeting the acetylcholine receptor (AChR-Abs) located at the postsynaptic membrane of the neuromuscular junction (1, 2). Thymomas are rare neoplasms originating from thymic epithelial cells, and their prognosis is unaffected by the concurrent presence of MG (3). Patients with TAMG typically present with generalized MG (gMG), characterized by muscle weakness affecting the limbs, neck and bulbar muscles (4–6).

Compared to other MG subtypes, TAMG is frequently more severe and associated with a higher risk of myasthenic crises and exacerbations (7, 8). These patients usually demonstrate a suboptimal response to standard treatments and have a poorer long-term prognosis, even after thymoma resection (9–11).

Conventional therapies for MG, including acetylcholinesterase inhibitors, corticosteroids and non-steroidal immunosuppressive therapies (ISTs), are effective for the majority of patients (12). However, approximately 10% of MG patients are classified as “refractory” to standard treatments, exhibiting an inadequate response to at least two ISTs other than corticosteroids (13–15). This finding underscores the urgent need for alternative therapeutic approaches.

Over the past five years, more targeted therapeutic strategies have been developed, such as eculizumab and ravulizumab — humanized monoclonal antibodies that inhibit the C5 protein. These therapies are currently approved for the treatment of acetylcholine receptor-positive generalized myasthenia gravis (AChR-gMG) (16–18) and have shown significant efficacy in symptom control. However, evidence supporting the use of complement inhibitors (CI) in TAMG remains limited, as these patients are frequently underrepresented or excluded from clinical trials, resulting in substantial gaps in treatment guidelines (19).

This study aims to report the real-world experience with CI therapy in patients with TAMG.

## 2 Methods

The study was approved by the Institutional Review Boards of the Fondazione Policlinico Universitario Agostino Gemelli IRCCS, the Cardarelli Hospital and the Sant’Andrea Hospital of Sapienza University (protocol ID 6743) and was conducted in accordance with the ethical standards laid down in the 1964 Declaration of Helsinki and its later amendments. All involved patients provided informed consent for the use of their anonymized medical records for research purposes.

### 2.1 Study population

We designed a multicentre, retrospective study, collecting data from patients with TAMG who attended the MG outpatient clinics at Fondazione Policlinico Universitario Agostino Gemelli IRCCS, Cardarelli Hospital and Sant’Andrea Hospital of Sapienza University, between June 2020 and November 2024.

Inclusion criteria were age over 18 years, a diagnosis of TAMG according to international guidelines (1, 20), and the initiation of treatment with either eculizumab or ravulizumab as part of clinical practice according to AIFA prescribing criteria, with a minimum follow-up of six months. Patients were treated with eculizumab if they had inadequate symptom control despite treatment with corticosteroids and at least two ISTs, with a Myasthenia Gravis-Activity Daily Living (MG-ADL) score of ≥6 and a Myasthenia Gravis Foundation of America (MGFA) classification of ≥III. Ravulizumab was prescribed for patients with an MG-ADL score of ≥6 and an MGFA classification of ≥IIb, who were unresponsive to at least one IST other than corticosteroids.

Data were collected from local databases and merged into a standardized database with predefined criteria for data categorization. We recorded demographics and clinical data, including age, sex, disease duration, MGFA clinical classification and Post Interventional Status (MGFA-PIS), thymoma histology and treatment, corticosteroid and ISTs dosages at baseline and during therapy, occurrence of myasthenic crises or exacerbations, MG-ADL and Quantitative Myasthenia Gravis (QMG) scores (21, 22).

### 2.2 Clinical outcomes analysis

Clinical outcomes were assessed using MG-ADL and QMG scores at baseline and at three, six and 12 months following the initiation of CI therapy. Responders were defined as patients achieving a reduction of at least 2 points in the MG-ADL score, and at least 3 points in the QMG score, compared to baseline. Moreover, we explored potential predictors of treatment response by adjusting outcome measures for baseline MG-ADL, baseline QMG, age, sex, and disease duration.

### 2.3 Steroid-tapering analysis

Steroid dosages were recorded at baseline and at three, six, and 12 months after CI therapy initiation. The percentage reduction in steroid doses from baseline at each time point were used to evaluate the steroid-sparing efficacy of CI therapy.

### 2.4 Control group of mg patients without thymoma

As a control group, we included randomly chosen patients with AChR-Abs-positive MG without thymoma, who were treated with CI according to international guidelines. These patients were recruited from the MG outpatient clinics at Fondazione Policlinico Universitario Agostino Gemelli IRCCS, Cardarelli Hospital, and Sant’Andrea Hospital of Sapienza University between June 2020 and November 2024.

### 2.5 Statistical analysis

The Shapiro-Wilk test was employed to verify the Gaussian distribution of the data. Categorical variables were presented as absolute frequencies and percentages, whereas continuous variables were summarized with medians and interquartile ranges (IQR). Pairwise comparisons between baseline and different time points (3, 6, and 12 months) within each treatment group were performed using the Wilcoxon signed-rank test. The Mann-Whitney U test was employed to perform pairwise comparisons between TAMG and control groups at baseline and at different time points. Fisher’s exact test was used to compare categorical variables between TAMG and control groups at baseline. A p-value <0.05 was considered statistically significant.

## 3 Results

We retrospectively included 23 patients with TAMG in this study: 13 treated with eculizumab and 10 with ravulizumab. Ten patients were followed at the MG outpatient clinic of Fondazione Policlinico A. Gemelli IRCCS in Rome, eight at Cardarelli Hospital in Naples and five at Sant’Andrea Hospital of Sapienza University in Rome. All patients were AChR-Abs positive and previously naïve to CI therapy.

### 3.1 Patient population

**Figure 1:**
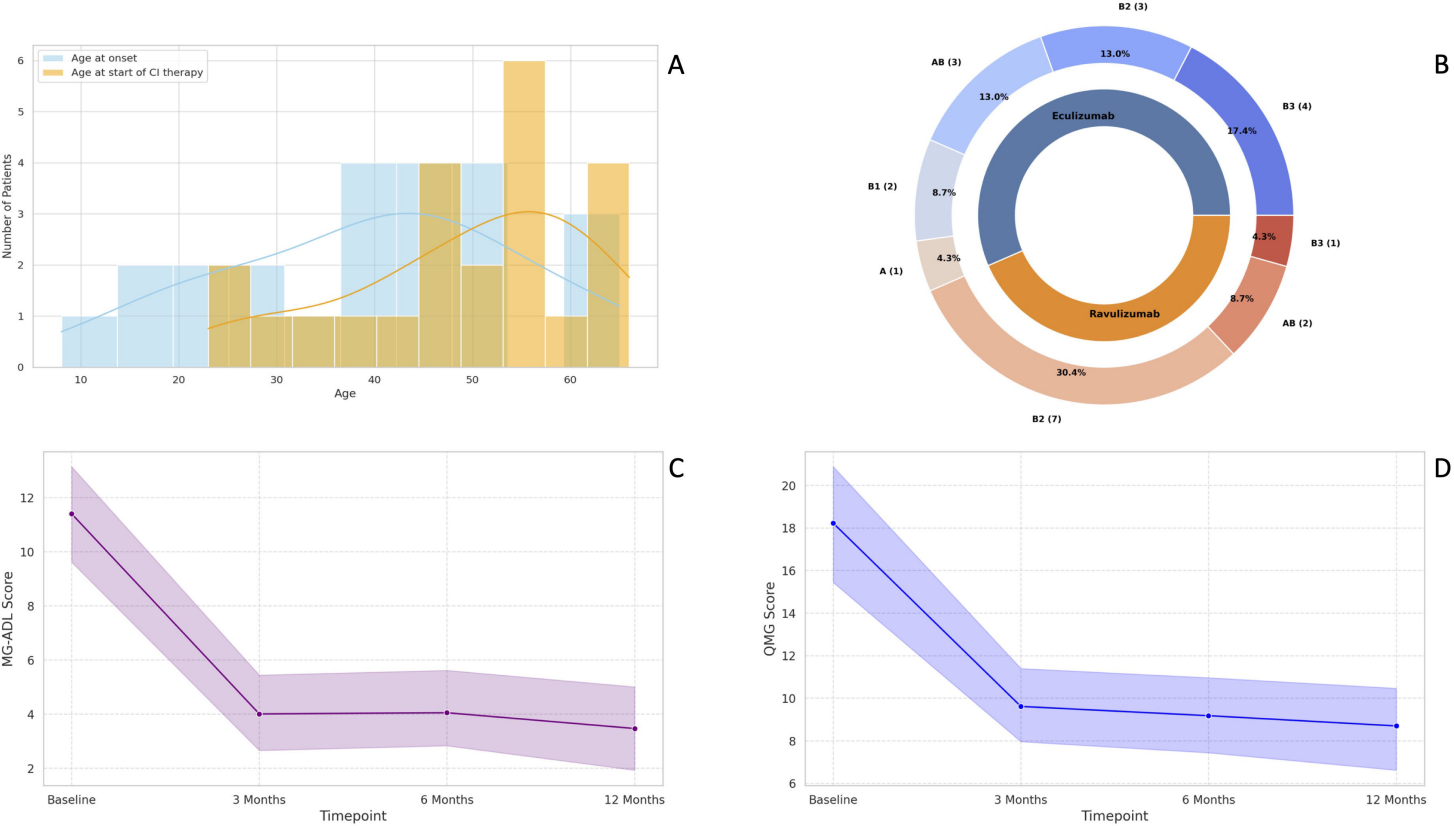
Baseline demographic characteristics of TAMG patients included in the study **(A)**; Distribution of WHO thymoma histological subtypes in our cohort **(B)**; Median change in MG-ADL scores at three, six- and 12-months follow-up **(C)**; Median change in QMG scores at three, six- and 12-months follow-up **(D)**. MG-ADL, Myasthenia Gravis-Activities of Daily Living; QMG, Quantitative Myasthenia Gravis; TAMG, Thymoma-associated Myasthenia Gravis; WHO, World Health Organization.

Baseline demographic and clinical characteristics of the included patients are reported in **Table 1**; **Figure 1A**.

**Table 1:** Demographic and clinical characteristics of study population. Continuous and categorical variables are reported as median (IQR) and number (%).

Number of TAMG patients	23
Eculizumab-treated patients	13
Ravulizumab-treated patients	10
Age at symptoms onset, years	42 (28–51)
Age at initiation of CI, years	53 (42–57)
Female sex, n (%)	11 (47.8)
Time from onset to CI, years	8 (1–14)
Refractory MG, n (%)	15 (65.2)
Max MGFA classification, n (%)	
I	0
II	0
III	10 (43.5)
IV	5 (21.7)
V	8 (34.8)
MG treatment at baseline, n (%)	
Oral corticosteroids	23 (100)
Azathioprine	9 (39)
Mycophenolate mofetil	2 (8.7)
Cyclosporine	2 (8.7)
Rituximab (within 6 months from baseline)	2 (8.7)
Chronic IV immunoglobulin	1 (4.35)
MGFA-PIS, n (%)	
CSR	0
PR	0
MM	6 (26.1)
I	15 (65.2)
U	1 (4.35)
W	0
E	1 (4.35)
Rescue therapy during CI, n (%)	
Plasmapheresis	1 (4.35)
Intravenous immunoglobulin	0
Thymoma (WHO classification), n (%)	
A	1 (4.35)
AB	5 (21.7)
B1	2 (8.7)
B2	10 (43.5)
B3	5 (21.7)
Thymoma (Masaoka-Koga stage), n (%)	
I	4 (17.4)
II	8 (34.8)
III	3 (13)
IV	3 (13)
n/a	5 (21.7)
Approach, n (%)	
Sternotomy	14 (60.9)
Minimally invasive surgery	3 (13)
n/a	6 (26)
Time from thymectomy to CI (n=21), years	8 (2.5-15)
Time from CI to thymectomy (n=2), months	2.5
Treatment other than surgery	
Chemotherapy	5 (22.7)
Radiotherapy	9 (39)
Thymoma recurrence	3 (13)

CI, Complement Inhibitors; CSR, Complete Stable Remission; E, Exacerbation; I, Improved; MG, Myasthenia Gravis; MGFA, Myasthenia Gravis Foundation of America; MGFA-PIS, Myasthenia Gravis Foundation of America – Post Intervention Status; MM, Minimal Manifestations; PR, Pharmacological Remission; TAMG, Thymoma-associated Myasthenia Gravis; U, Unchanged; W, Worsened; WHO, World Health Organization.

Patients had a median age of 53 years (IQR: 42-57) at baseline, 11 out of 23 (47.8%) were female, and all were Caucasians. All patients underwent a minimum six-months follow-up, while 14/23 (60.9%) had an available follow-up of 12 months.

Most patients (91.3%) started CI therapy after thymectomy, with a median interval of eight years (IQR: 2.5-15) after surgery. Conversely, two patients achieved sufficient clinical stabilization on CI to undergo thymectomy thereafter, with a median interval of 2.5 months.

The most common thymoma histological subtype was the WHO type B2, detected in 10 out of 23 patients (43.5%), followed by AB type and B3 type, identified in five patients each (21.74%) (**Figure 1B**). Only one patient had an ongoing thymoma recurrence at the time of CI therapy initiation.

Regarding safety, no infections were reported during treatment up to the last available follow-up.

### 3.2 MG-ADL

The median MG-ADL score significantly improved from 11 (IQR: 7-15) at baseline to 3 (IQR: 2-5) at three months and 4 (IQR: 1-5) at six months (both p < 0.001). At the six-month follow-up, 21 patients (91.3%) were classified as MG-ADL responders. Both non-responders were treated with ravulizumab and had thymoma histology classified as the WHO type B2. Neither received adjuvant therapy nor experienced thymoma recurrence. One of these two non-responders had a clinical exacerbation six months after initiating CI, requiring plasma exchange and an increase in daily steroid dosage (from 10 mg to 37.5 mg). At the 12-month follow-up, this patient showed significant improvement and was reclassified as a “late-responder” to CI therapy. However, the potential contribution of plasma exchange and increased steroid dosage to the observed improvement cannot be ruled out. Among the 14 patients with available 12-month follow-up data, the median MG-ADL score remained stable at 3 (IQR: 0–6) (**Figure 1C**).

### 3.3 QMG

The median QMG score improved from 16 (IQR: 14-22) to 10 (IQR: 7-14) at three months and remained stable at 10 (IQR: 5-11) at six months (both p < 0.001). After six months of CI therapy, 21 patients (91.3%) were classified as QMG responders. Among the two non-responders, one was treated with eculizumab and the other with ravulizumab. The eculizumab-treated non-responder had thymoma histology classified as the WHO AB subtype and was eventually reclassified as a “late-responder”, showing a 4-point improvement in QMG score after 12 months of CI therapy. The ravulizumab-treated non-responder had thymoma histology of the WHO B2 subtype, was treated with adjuvant chemotherapy and previously experienced thymoma recurrence. No further follow-up data were available for this patient, and a delayed response to CI therapy could not be ruled out. At 12 months, the median QMG score for the 14 TAMG patients with available follow-up clinical data further decreased to 8.5 (IQR: 5-10.5) (**Figure 1D**).

### 3.4 Predictors of treatment outcome

Our analysis showed that none of the examined covariates (baseline MG-ADL, baseline QMG, age, sex, and disease duration) significantly influenced changes in MG-ADL or QMG scores. Given the lack of significant associations, adjusted regression models did not provide additional explanatory value beyond unadjusted estimates.

### 3.5 Concomitant medications

At baseline, all patients were receiving corticosteroids, with a median daily dosage of 25 mg (IQR: 18.75-50). Moreover, 14 patients were on non-steroidal ISTs, including azathioprine (n=9), mycophenolate mofetil (n=2), cyclosporine (n=2), and rituximab within six months of CI initiation (n=2). The median treatment duration with non-steroidal ISTs before CI initiation was 8 years (IQR: 0-18). A total of 20 patients (87%) achieved a reduction in oral corticosteroid dosage. The percentage reduction in steroid dosage was 25.62% at three months (p < 0.001), increasing to 36.37% (p < 0.001) at six months after the first CI administration. Among the 14 patients with a 12-month follow-up, the median steroid dosage was 10 mg (IQR: 5-15.6). By the last available follow-up, three patients had discontinued steroid use entirely, two had withdrawn azathioprine, and one had discontinued cyclosporine. One patient initiated mycophenolate mofetil during CI therapy due to persistent symptom fluctuations, despite initial clinical improvement.

Among the two MG-ADL non-responders, one patient reduced the daily steroid dosage from 20 to 15 mg, while the other experienced a clinical deterioration, necessitating an increase in steroid dosage from 10 to 37.5 mg at 6 months, followed by a gradual taper to 25 mg at 12 months. Regarding QMG non-responders, one patient maintained a stable steroid daily dosage of 10 mg, while the other progressively reduced steroid use from 50 to 35 mg at 6 months, and further to 25 mg after one year, as he was reclassified as a QMG late responder to CI therapy.

### 3.6 MGFA-PIS

The MGFA-PIS (23) classification demonstrated significant improvement following CI therapy. At the last available follow-up, 15 patients (65.23%) were classified as having an “improved” status, while six patients (26.1%) achieved “minimal manifestation” status. One patient remained “unchanged” in MGFA-PIS, while another experienced a clinical exacerbation, requiring rescue therapy with plasma exchange and an increase in daily steroid dosage. No myasthenic crises were reported during the follow-up period.

### 3.7 Comparisons between TAMG patients and MG patients without thymoma

We retrospectively included 22 MG patients without thymoma in the control group, 19 treated with eculizumab and three with ravulizumab. Nine patients were followed at the MG outpatient clinic of Fondazione Policlinico A. Gemelli IRCCS in Rome, nine at Cardarelli Hospital in Naples and four at Sant’Andrea Hospital of Sapienza University in Rome. All were AChR-Abs positive and naïve to CI therapy.

The TAMG and control groups had comparable demographic and clinical characteristics at baseline, including sex, age at symptom onset, age at initiation of CI therapy, disease duration, disease severity (maximum MGFA score), median MG-ADL and QMG scores, median steroid dosage.

Pairwise comparisons using the Mann-Whitney U test revealed no statistically significant differences between the two groups in median MG-ADL score reduction (p = 0.41 at 3 months, p = 0.71 at 6 months, and p > 0.99 at 12 months), median QMG score reduction (p = 0.71 at 3 months, p = 0.58 at 6 months, and p = 0.81 at 12 months), and steroid tapering (p = 0.88 at 3 months, p = 0.87 at 6 months, and p = 0.99 at 12 months) at any time point.

**Figure 2:**
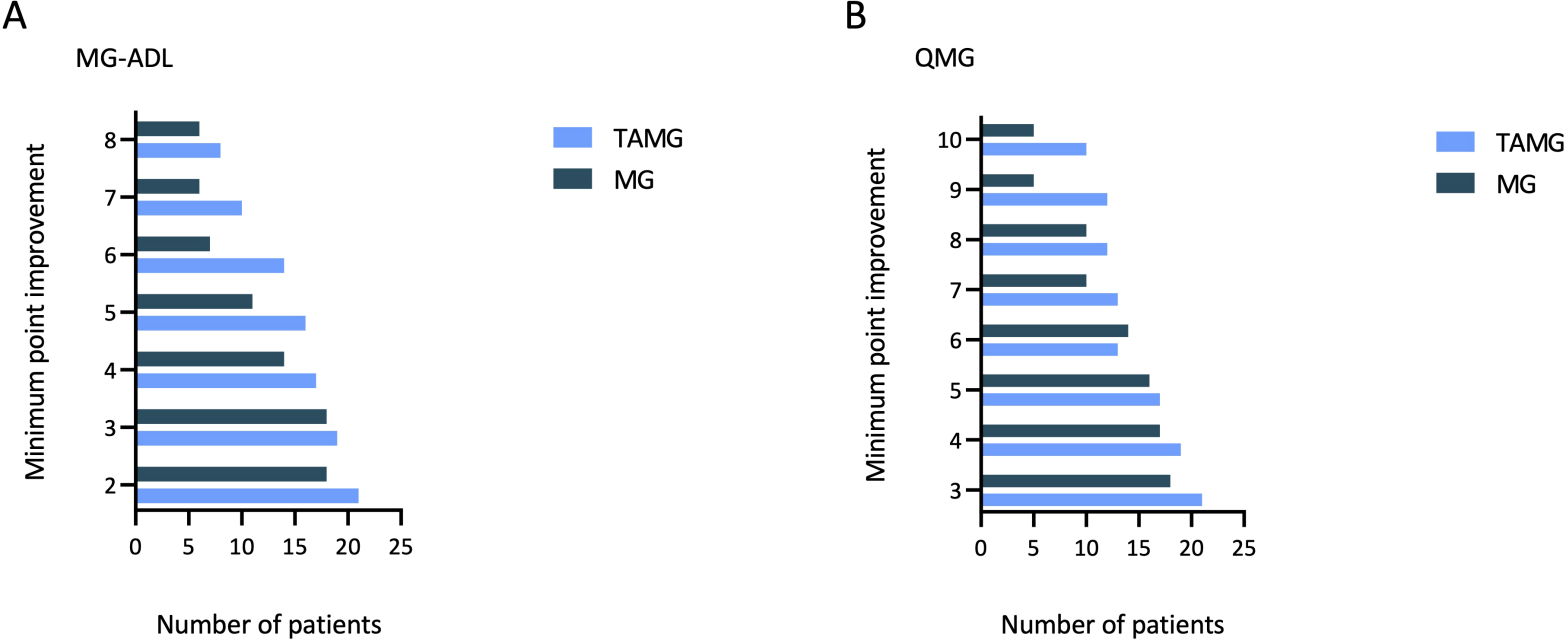
Bar chart for the distribution of minimal point improvement in MG-ADL score from baseline to 6-months follow-up for TAMG patients and MG patients without thymoma **(A)**; Bar chart for the distribution of minimal point improvement in QMG score from baseline to 6-months follow-up for TAMG patients and MG patients without thymoma **(B)**. MG-ADL, Myasthenia Gravis-Activities of Daily Living; QMG, Quantitative Myasthenia Gravis; TAMG, Thymoma-associated Myasthenia Gravis.

At the six-month follow-up, three control patients (13.64%) were classified as non-responders based on both MG-ADL and QMG scores, while one was a non-responder for MG-ADL and another for QMG. Overall, the responder rate to CI therapy for MG patients without thymoma was 81.8% according to MG-ADL and to QMG scores (**Figures 2A, B**).

At the last available follow-up, the MGFA-PIS classification in the control group was as follows: 12 patients (55%) were classified as “improved”, four (18%) as “minimal manifestations”, and three (13.64%) as “unchanged”. One patient experienced a clinical exacerbation that required rescue therapy with two cycles of intravenous immunoglobulins and subsequently withdrew from CI therapy after six months of treatment. Additionally, “complete stable remission” and “pharmacological remission” were achieved by one patient each.

## 4 Discussion

In this real-world study, we evaluated the efficacy of CI therapy in patients with TAMG, a subgroup of MG characterized by challenging management and suboptimal response to conventional treatments. Our findings demonstrated a median reduction of 7 points in the MG-ADL score at six months, with a responder rate of 91.3%. Similarly, the QMG score showed a median improvement of 6 points at six months, with the same responder rate of 91.3%. Interestingly, the two MG-ADL non-responders were classified as QMG responders, and vice versa. These discrepancies likely reflect the differences in the assessment methods: MG-ADL reflects patient-reported symptoms over the previous week, while QMG is a physician-reported evaluation conducted at a single time point.

Among the four non-responders, three had thymoma histology classified as the WHO B2 subtype, which is not unexpected given its higher prevalence in our cohort. None of the non-responders experienced thymoma recurrence during treatment. While thymoma recurrence in TAMG is known to exacerbate clinical symptoms, its impact on CI therapy response remains unknown. Notably, in our cohort, one patient had an ongoing thymoma recurrence at CI initiation, and he was classified as a “responder” based on both MG-ADL and QMG scores.

The findings of this study align with the efficacy outcomes reported in the phase III REGAIN trial of eculizumab and the phase III CHAMPION MG trial of ravulizumab, as well as their respective open-label extension studies (16, 17, 24). While patients with a history of thymoma were excluded from REGAIN, they were eligible for CHAMPION MG if they had no active or untreated thymoma and no evidence of recurrence for at least five years prior to screening. Compared to these trials, our findings indicated a more robust and consistent response to CI therapy in TAMG patients, characterized by greater reductions in both MG-ADL and QMG scores. These results are consistent with Japanese post-market surveys and a recent American real-world study (25, 26). They also partially align with another Japanese real-world study, which reported milder improvements in this subgroup (27). Conversely, a recent German cohort study observed clinically meaningful responses to CI therapy in 61% of patients according to MG-ADL and 51% according to QMG. However, only 16% of the included patients had TAMG, and no specific analysis was conducted for this subgroup (28).

The notable improvements observed in our cohort may be partially explained by the high baseline disease burden, as reflected by elevated MG-ADL and QMG scores, which likely provided greater room for measurable clinical gains following treatment. To partially address this limitation, we compared the reductions in median MG-ADL and QMG scores, along with steroid tapering, between TAMG patients and a control group of MG patients without thymoma. No statistically significant differences were observed at any time point. Moreover, the marked clinical benefit in TAMG population were further supported by the MGFA-PIS classification, which demonstrated significant improvement at the last available follow-up for most patients. Only one non-responder experienced clinical exacerbation, requiring plasma exchange after six months, while no myasthenic crises were observed throughout the study period.

Another possible explanation for the increased efficacy of CI therapy observed in TAMG is that the greater disease severity and burden in this subgroup may be associated with a more pronounced complement activation and a higher degree of complement-mediated damage at the neuromuscular junction. However, further studies are needed to analyse the contribution of complement-mediated mechanisms in TAMG and to optimize targeted therapeutic strategies for these patients.

Most patients (91.3%) underwent thymectomy before CI therapy, raising the possibility that surgical intervention contributed to treatment outcomes. However, given the median interval of 8 years between thymectomy and CI therapy initiation, and the poor disease control at baseline, the direct role of thymectomy on disease stabilization remains unclear. Conversely, two patients underwent thymectomy after achieving sufficient clinical stabilization on CI therapy, with a median interval of 2.5 months between CI initiation and surgery. In these cases, thymectomy may have contributed to maintaining long-term clinical stability.

Steroid-tapering was achieved in the majority of TAMG patients, with many maintaining clinical stability or demonstrating improvement despite reductions in concomitant ISTs. These findings suggest the potential of CI therapy as a steroid-sparing option for this challenging MG subgroup, possibly mitigating the long-term side effects associated with prolonged corticosteroid use (29).

This study has several limitations, including a small sample size and a relatively short follow-up period, due to the recent approval of CI therapy. QMG assessments were performed by different raters, and changes in steroid dosages were not standardized but rather left to the discretion of the treating clinicians. Additionally, all patients were receiving corticosteroids at baseline, and 14 patients had been on ISTs for a median of 8 years before CI therapy initiation as an “add-on”, suggesting that long-term immunosuppression may have been contributed to the observed clinical improvements, especially considering its cumulative effects over time. Further research with larger patient cohorts and extended follow-up periods is needed to fully evaluate the efficacy of CI therapy and optimize its combination with standard immunosuppressive treatments in patients with TAMG, a subgroup that remains challenging and difficult-to-treat.

## Data Availability

The raw data supporting the conclusions of this article will be made available by the authors, without undue reservation.
